# Fracture Dislocation at the Level of C6-C7: A Case Report and Literature Review

**DOI:** 10.7759/cureus.34675

**Published:** 2023-02-06

**Authors:** Nouf A Altwaijri, Rami Barakat, Hani Alharbi, Norah Romaih, Abdullah Aldhafeeri

**Affiliations:** 1 Orthopaedic Surgery, King Saud Medical City, Riyadh, SAU; 2 Orthopaedics, King Saud Medical City, Riyadh, SAU

**Keywords:** cervical fracture, cervical spine fracture, c6-7 fracture dislocation, cervical spine, fracture dislocation of cervical spine

## Abstract

Fractures of the cervical spine can cause devastating long-term effects on patients. Spinal cord injuries can occur in up to 50% of cases in association with cervical spine fractures. Therefore, it is vital and of utmost importance to recognize cervical spine injuries early on to avoid the exacerbation of an existing injury and its detrimental effects on the patients. We report a case of a C6-C7 fracture dislocation with an associated neurological insult that improved dramatically following fixation and rehabilitation. Unfortunately, patients with this presentation may have long-term neurological insults rather than regain normal function; however, our case notes the importance of prompt intervention and its effect on the outcome.

## Introduction

Cervical spine fractures can cause long-term effects on patients where almost 50% of spinal cord injuries have been reported in relation to cervical spine fractures [1-10]. Therefore, it is vital to identify cervical spine injuries as soon as possible to avoid exacerbating an existing injury and its possible long-term effects on the patients. Clayton et al. examined the possible predictors of cervical spine injuries and found that Motor Vehicle Collision (MVC), falls, age <40, pelvic fractures, and an Injury Severity Score (ISS) of >15 are significant individual predictors of cervical spine injury. Interestingly, neither facial fracture nor head injury alone correlated with a higher risk of cervical spine injury [11]. We report a case of a C6-C7 fracture dislocation with an associated neurological insult that improved dramatically following fixation and rehabilitation. We present the following case in accordance with the CARE reporting checklist.

## Case presentation

**Figure 1:**
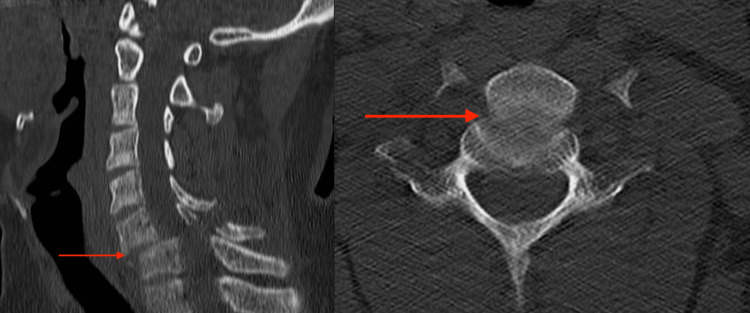
CT cervical axial (right) and sagittal (left) cuts showing fracture dislocation of C6-C7

Our patient is a 41-year-old Saudi man with a history of a motor vehicle accident where he hit a camel on a desert road at the speed of 110 km/hr in September 2019. He presented to our emergency department complaining of severe neck pain and limitation of movement and was on C-collar. He had no previous significant medical or surgical history. After primary and secondary surveys, his physical examination revealed a right upper limb neurological deficit in the form of shoulder numbness, and a C5-T1 power of 3/5; whereas, his left upper limb and bilateral lower limbs were intact neurologically. He was controlling his sphincters and had no evident vascular injury. Images were taken for the patient upon admission; radiographs and CT images showed translation of the cervical at the level of C6-C7 of more than 75% anteriolisthesis (Figure 1).

**Figure 2:**
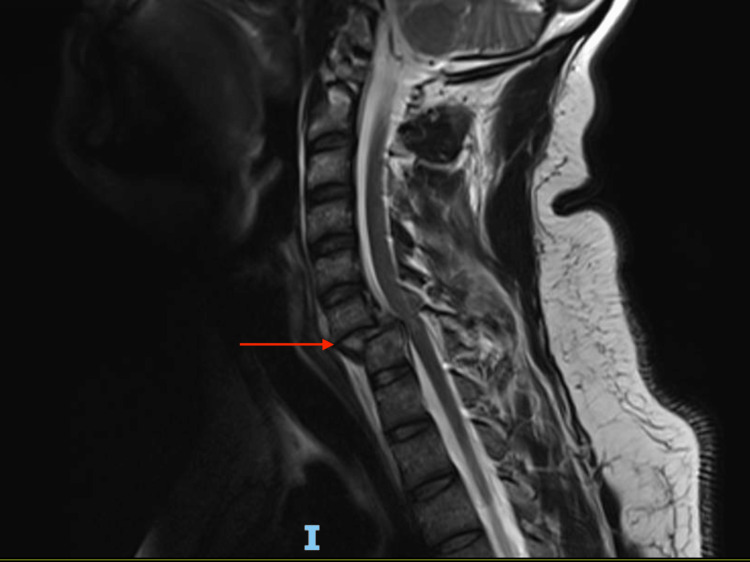
MRI cervical showing C6-C7 fracture dislocation

**Figure 3:**
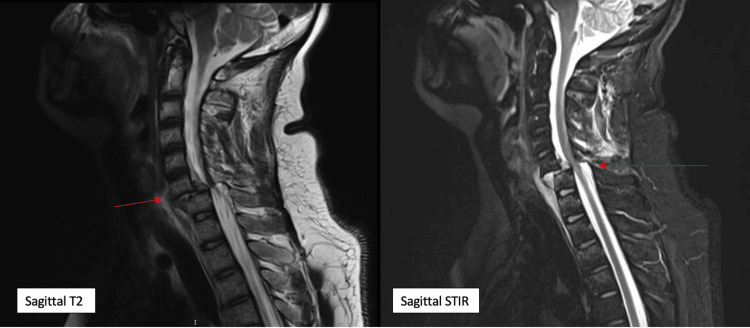
MRI sagittal T2 and STIR views showing C6-C7 fracture dislocation STIR: Short Tau Inversion Recovery

No other injuries or fractures were noted. MRI performed showed a traumatic disc at the same level compromising the spinal canal with posterior disruption of the ligamentous complex (Figures 2-3).

**Figure 4:**
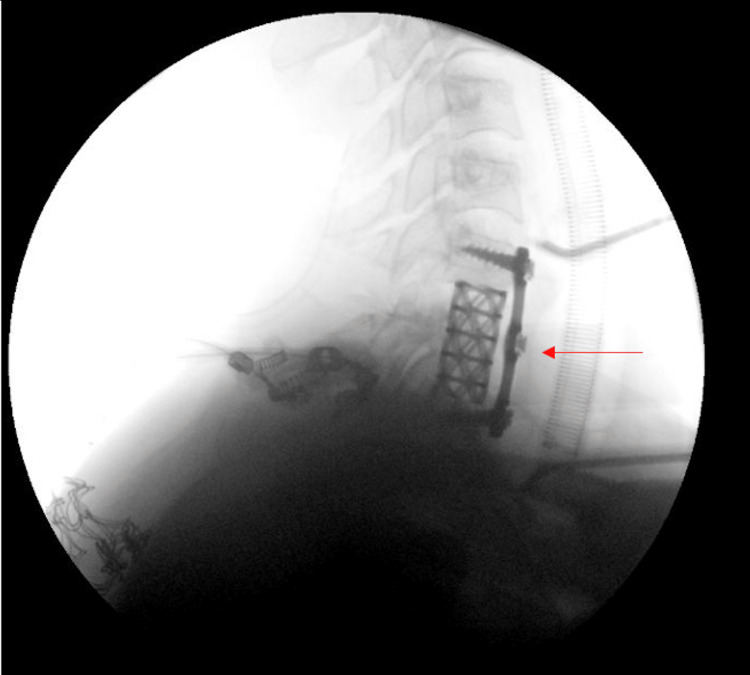
Intraoperative lateral view of posterior bilateral facetectomy and corpectomy effusion at the level of C7; cage and plate fixation from C6-T1

**Figure 5:**
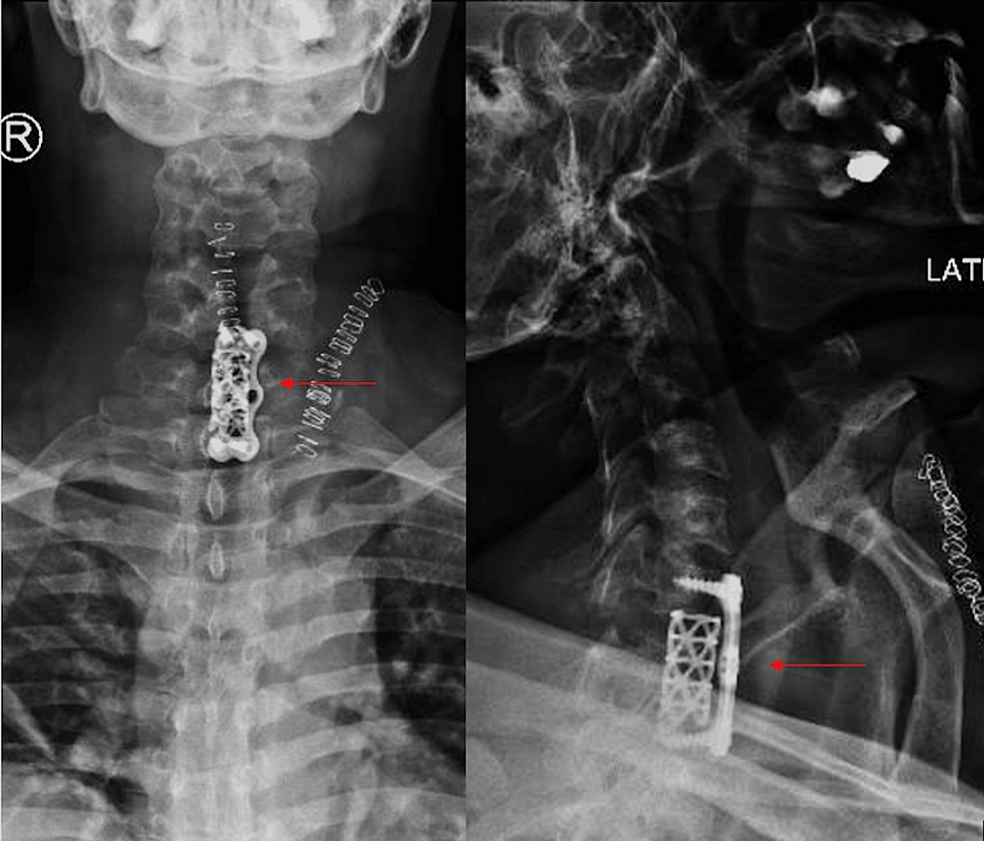
Anterior-posterior and lateral views of fixation post operatively

The patient remained on C-collar, optimized and prepared for surgery. Moreover, the patient was observed under the care of the ICU, and was pushed to the operating room urgently in under 24 hours. Patient was put in prone position, prepping and draping were done in a sterile manner. The surgery began posteriorly, utilizing a posterior midline incision where facetectomy was done completely in the posterior part of C7. Afterwards, the patient was positioned supinely and draped again for the anterior approach for complete corepctomy and proper decompression at the level of C7 with mesh plating of C6-C7 and bone grafting, followed by close observation under the ICU for the next couple of days (Figures 4-5).

**Figure 6:**
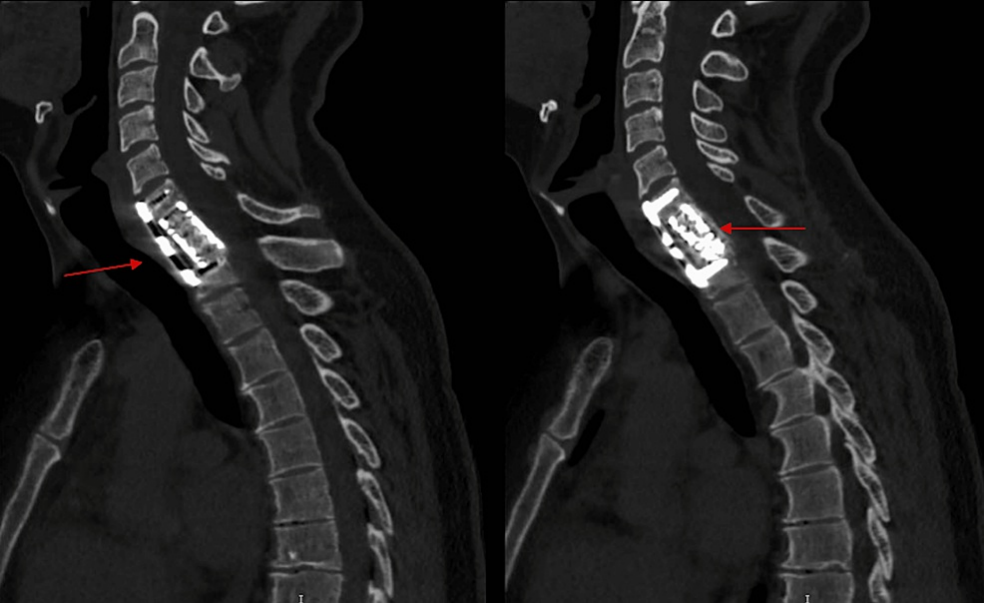
CT Cervical sagittal views at two years follow up

**Figure 7:**
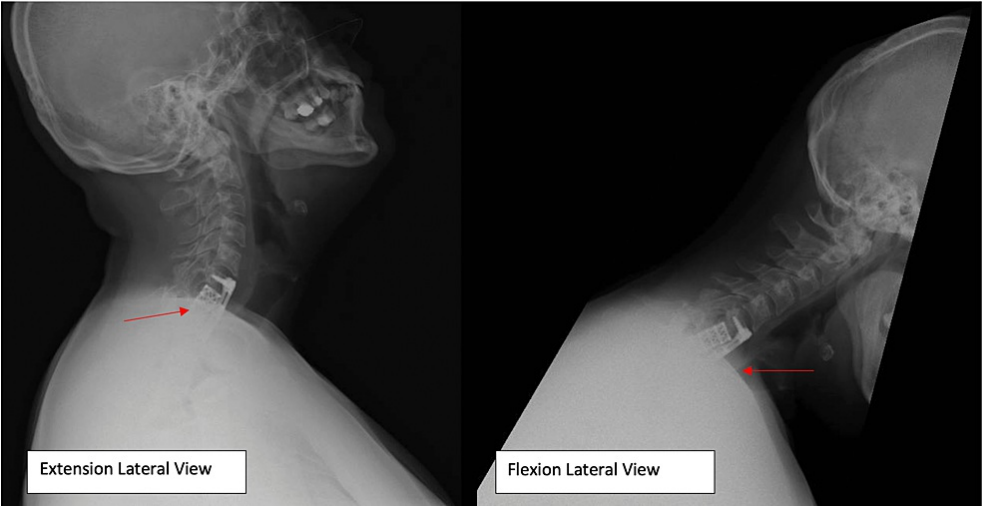
Latest lateral X-rays at the last follow up; flexion and extension views

The patient had multiple visits and follow up with evident improvement in his condition as well as gradual improvement in power and pain in the right upper limb. At his two years follow up, his neurological status was back to normal; his power is now 5/5, and sensation 2/2 all over the upper and lower limbs. He was able to fully get back to work. His radiographs were excellent with a normal kyphotic degree (Figures 6-7).

## Discussion

We presented a case of grade 4 anterolisthesis at the level of C6-7 with incomplete right upper limb paralysis that regained normal neurological status at the latest follow up. 

It is noted that cervical fracture dislocations most commonly occur at the level of C6-7 and C5-6 following trauma [12,13]. Falls seems to be the most common mode of injury in several papers [14,15]. Fredø et al. also noted a male predominance in cervical fractures, in addition to a mortality rate of 10% in the Norwegian population [14]. Axial load or a large compressive force applied to the top of the head is the major mechanism of serious cervical injury, more so when the neck is in slight flexion given that the spine is out of its normal lordotic alignment leading to improper distribution of force to the thorax, where the musculature cannot aid in absorbing the force due to the cervical spine being in a straight line due to flexion [16].

Adeolu et al. studied the effectiveness of closed reduction of cervical spine injuries using cervical traction and noted an improvement in the neurological function of 18.9% whilst the rest remained neurologically the same. He noted the causes of failure to be locking facets most commonly, old injuries, new-onset or worsening pain, and over-distraction. In addition, the complications of reduction in a closed manner were over-distraction most commonly, tong pull-out, new-onset or worsening pain, and finally skull perforation. However, they concluded that satisfactory reduction can be achieved in patients with cervical spine injuries and significant malalignment [17].

The sooner the spinal cord is stabilized with decompression of the injured spinal cord, the higher the chance of recovery [18,19]; reducing the fracture or dislocation will bring the vertebral canal back to its normal form and dimension leading to spinal cord decompression [18,20]. Abdelgawaad et al. [21] evaluated the efficacy of the anterior only approach for C F4 (AO classification) traumatic subaxial cervical spine injuries and concluded that cervical traumatic instability can be efficiently managed surgically with anterior decompression and fusion. They recommend the anterior approach in cases with neurological deficits, in patients with comorbidities, or when a short operative time is preferred. Zhou et al conducted a prospective study evaluating cervical pedicle screw fixation for fracture dislocation of the lower cervical spine and found that all participants had achieved solid bony fusion at six months follow up in addition to stable fixation of the related segments. In their paper, patients with complete spinal cord injury showed no improvement in neural function except for mild alleviation of pain and numbness; whereas patients with incomplete injury showed a grade or two on the Abbreviated Injury Scale (AIS) classification [22].

Recent guidelines necessitate surgery to achieve reduction and stabilization of the cervical spine and insure spinal cord decompression due to the instability naturally associated with dislocations [18,23]. Interestingly, not all fracture dislocations are managed immediately. According to Miao et al., treating old fracture dislocation of the lower cervical spine can be managed with completed decompression, immediate and long-term anterior cervical column support sequence, and physiological curvature of the cervical vertebra, in addition to restoring nerve function using anterior partial corpectomy, titanium mesh fusion, and internal fixation. When there are locked facet joints or posterior structures invading the vertebral canal, the combined anterior and posterior approaches should be performed in order to achieve better results [24]. 

It is, unfortunately, possible for some patients with a such presentation to end up with a long-term neurological deficit rather than regain normal function which is demonstrated in the cases found within the literature review in Table 1. It is therefore vital to approach such cases with a focused and efficient approach in order to maximize the possible benefits for the patients. 

**Table 1:** Literature review of similar cases of fracture dislocation AIS: Abbreviated Injury Scale; CSF: cerebrospinal fluid; ASIA:  American Spinal Injury Association Impairment Scale; MO: myositis ossificans; NSAIDs: nonsteroidal anti-inflammatory drugs; ACCF: anterior cervical corpectomy and fusion.

Author	A	G	Fracture Dislocation	Mechanism	Presentation	AIS [25]	Management	Outcome
Müller et al. [26]	37	F	C4/5	hit by a horse	complete paraplegia	Grade A [admission] to grade B [postoperatively].	Emergency reduction 120 minutes post trauma followed by a standard anterior decompression, discectomy, and fusion.	At one year follow up, she became ambulatory without walking aids and restarted horse riding. Her AIS score is grade D.
Roda et al. [27]	-	-	C2/3	Hangman’s fracture	No dysfunction of spinal cord	-	Conservative with immobilization	Solid healing of fracture and good alignment of the cervical spine.
Li et al. [28]	39	F	C5/6	accidental strangulation	She developed paravertebral abscesses, cutaneous fistulas and oesophageal perforation	-	Conservative by halo vest due to poor general condition. The oesophagus-cutaneous fistula was managed with enteral tube feeding and repeated local care.	At her sixth year follow-up she survived with complete C5 tetraplegia.
Pinter et al. [29]	50	M	atypical hangman fracture of C2 and right-sided facet fracture of C4 with traumatic spondylolisthesis at C4/5.	Fall	2/5 strength in the right deltoid and biceps and 3/5 strength in the left deltoid and biceps with no motor or sensory function distal to the C5 level.	-	C2–C5 anterior cervical discectomy and fusion followed by a C3–C5 posterior instrumented fusion.	2 years post op showed 4/5 strength in deltoids and biceps and 2/5 strength in right wrist extension. Solid arthrodesis shown on flexion–extension radiographs
Zan et al. [30]	36	M	C7/T1	Fall	sensory and motor disturbance for 12 hours. Known case of Ankylosing Spondylitis	-	Posterior C5-T3 cervical instrumentation and fusion + anterior and posterior fixation followed by open reduction, internal fixation of C7-T1 fracture and cervical plating anteriorly with an iliac crest autogenous bone grafting	Post operatively, muscle strength of 3/5 in the upper extremities and 3+/5 in the lower extremities and pain subsided.
Baker et al. [31]	59	M	C6/7	singlevehicle accident	Marked cervical muscle spasm. Unremarkable motor examination except for minimal right triceps weakness, no sensory deficit. Normal deep tendon reflexes except for right triceps which was slightly decreased.	-	Started with failed skeletal traction followed by manual traction under fluoroscopic control which achieved partial reduction, skeletal traction was applied after and continued with 7 lb weight. Followed by halo vest application.	Two years after his injury, his cervical spine remains in good alignment with normal neurological examination.
Bhatia et al. [32]	45	M	C6/7	Fall from a roof top and loss of consciousness	Severe neck paining marked restriction of neck movements. No neurological deficit.	-	30 pounds skeletal traction achieved a partial reduction followed by posterior fusion with wire and bone and excision of C7 body, fixation and bony fusion with an iliac crest graft with wire loops, utilizing an anterolateral operative approach.	No neurologica deficits noted a week after, patient discharged on minerva jacket.
Raudenbush et al. [33]	30	M	C2 fracture-dislocation with C1 ring and occipital condyle avulsion fracture	Motorcycle accident	Incomplete C2 spinal cord injury and poly trauma with multiple orthopedic injuries.	-	C1 - C4 posterior instrumented spinal fusion performed. Cerebrospinal fluid seen, but no decompression done. No improvement 6 weeks post op. X rays showed acceptable alignment of the spine and appropriate position of the spinal implants. The patient remained hospitalized for over 3 months. 12 weeks later, he started complaining of neck pain which turned out to be MO of the right longus coli muscle which was managed by passive stretching, NSAIDs, antispasmodics, and a rigid cervical collar as tolerated. Moreover, the patient complained of persistent headaches; CT head showed chronic subdural hematomas, which were attributed to a persistent CSF leak at the site of the fracture-dislocation which was managed with a percutaneous blood patch.	8 months post operatively, he noted continued neck and neuropathic pain, however, he stated that the pain was better in comparison to his initial post-operative period.
Yang et al. [34]	45	M	C6/7	involved in an architectural accident	Presented with neck pain for 4 months	D	Posterior lateral mass screw fixation of C5,7 (bilaterally) and C6 (left side) in a different institute followed by rehabilitation. However, 4 months post op, neck pain and muscle weakness in all right side limbs were still present. Examination showed hypoesthesia and myodynamia of the right-side limbs weakness 4/5. Hoffman and Babinski signs were negative. The X-rays showed the re-dislocation of C6/7 confirmed by CTt. Revision surgery of ACCF with nanohydroxyapatite/polyamide 66 composite fulfilled with vertebral autograft plus anterior plate was done.	3 months post operatively, images showed the good position of the implant and bony fusion. The patient’s neck pain subsided, and neurological function restored to ASIA E grade.
Shiina et al. [case 1] [35]	51	M	C5/6	Motor vehicle accident	Presented with shock and was intubated.Diagnosed with C5/6 fracture dislocation and fracture of the right lateral mass of C6	-	Manual reduction failed; traction up to 10 kg applied and failed. Immobilization in a halo vest was done. Due to respiratory function deterioration, tracheotomy was performed 1 month after the injury	2 months later, callus formation on x rays was noted and the halo vest was changed to a Philadelphia collar. No improvement of paralysis was observed. He died of pneumonia at another hospital 3 months later.
Shiina et al. [case 2] [35]	32	M	C5/6	His left mandible was smashed into a hanging steel sheet while working at a construction site.	Muscle weakness noted below the right and left brachial biceps and numbness of both upper limbs		Immobilization in a halo vest was applied on the day after injury. The mandible contusion healed without deep infection. 2 weeks later, posterior fusion and reduction followed by an autologous bone grafting followed by anterior decompression and fixation. Immobilization in a halo vest continued postoperatively because the patient was young and active. 2 weeks later, halo vest was changed to Philadelphia collar.	Both the degree and range of numbness decreased over time, and muscle strength in both upper limbs gradually improved. Bone union was confirmed 6 months post op. Paralysis in the fingers of both hands remained, but he was able to return to work.
Botolin et al. [36]	54	M	C7/T1	Fell over the handlebars of his racing bicycle	Presented neurologically intact. Initial CT showed a right sided C7/T1 fracture-dislocation, and a right-sided C6 and C7 traumatic laminotomy, and a left-sided C6/C7 perched facet.	-	Halo vest application without closed reduction, subsequent MRI revealed an acute traumatic C7/T1 disc herniation, therefore, definitive three-stage anterior-posterior-anterior spinal decompression, realignment, fixation and fusion C4-T2 were done in one session.	The patient recovered well and retained full neurological function.

## Conclusions

In conclusion, cervical spine fractures remain a devastating injury, especially when associated with dislocations, however, the timing of intervention and mode of intervention can drastically change the outcome; the return to complete function is a good possibility with adequate surgical decompression and stabilization of the spine.

## References

[1] Yadollahi M, Paydar S, Ghaem H (2016). Epidemiology of cervical spine fractures. Trauma Mon.

[2] Hu R, Mustard CA, Burns C (1996). Epidemiology of incident spinal fracture in a complete population. Spine.

[3] DesPlaines DesPlaines (1990). Committee on Injury Scaling Association for the advancement of automotive medicine. The Abbreviated Injury Scale. https://scholar.google.com/scholar?q=intitle:Committee%20on%20Injury%20ScalingAssociation%20for%20the%20advancement%20of%20automotive%20medicine.%20The%20abbreviated%20injury%20scale#d=gs_cit&t=1673733385305&u=%2Fscholar%3Fq%3Dinfo%3A3I-H9R6oj2kJ%3Ascholar.google.com%2F%26output%3Dcite%26scirp%3D0%26hl%3Den.

[4] Bayless P, Ray VG (1989). Incidence of cervical spine injuries in association with blunt head trauma. Am J Emerg Med.

[5] Sterne JA, White IR, Carlin JB (2009). Multiple imputation for missing data in epidemiological and clinical research: potential and pitfalls. BMJ.

[6] Thompson WL, Stiell IG, Clement CM, Brison RJ (2009). Association of injury mechanism with the risk of cervical spine fractures. CJEM.

[7] Soicher E, Demetriades D (1991). Cervical spine injuries in patients with head injuries. Br J Surg.

[8] Mulligan RP, Friedman JA, Mahabir RC (2010). A nationwide review of the associations among cervical spine injuries, head injuries, and facial fractures. J Trauma.

[9] Pedram H, Reza ZM, Reza RM, Vaccaro AR, Vafa RM (2010). Spinal fractures resulting from traumatic injuries. Chinese J Traumatol.

[10] Varma A, Hill EG, Nicholas J, Selassie A (2010). Predictors of early mortality after traumatic spinal cord injury: a population-based study. Spine (Phila Pa 1976).

[11] Clayton JL, Harris MB, Weintraub SL (2012). Risk factors for cervical spine injury. Injury.

[12] Okereke I, Mmerem K, Balasubramanian D (2021). The management of cervical spine injuries-a literature review. Orthop Res Rev.

[13] Cusick JF, Yoganandan N (2002). Biomechanics of the cervical spine 4: major injuries. Clin Biomech.

[14] Fredø HL, Rizvi SA, Lied B, Rønning P, Helseth E (2012). The epidemiology of traumatic cervical spine fractures: a prospective population study from Norway. Scand J Trauma Resusc Emerg Med.

[15] Malik SA, Murphy M, Connolly P, O'Byrne J (2008). Evaluation of morbidity, mortality and outcome following cervical spine injuries in elderly patients. Eur Spine J.

[16] Bailes JE, Petschauer M, Guskiewicz KM, Marano G (2007). Management of cervical spine injuries in athletes. J Athl Train.

[17] Adeolu AA, Ukachukwu AK, Adeolu JO, Adeleye AO, Ogbole GI, Malomo AO, Shokunbi MT (2019). Clinical outcome of closed reduction of cervical spine injuries in a cohort of Nigerians. Spinal Cord Ser Cases.

[18] Marcon RM, Cristante AF, Teixeira WJ, Narasaki DK, Oliveira RP, de Barros Filho TE (2013). Fractures of the cervical spine. Clinics (Sao Paulo).

[19] Fehlings MG, Vaccaro A, Wilson JR (2012). Early versus delayed decompression for traumatic cervical spinal cord injury: results of the Surgical Timing in Acute Spinal Cord Injury Study (STASCIS). PLoS One.

[20] Miranda TA, Vicente JM, Marcon RM, Cristante AF, Morya E, Valle AC (2012). Time-related effects of general functional training in spinal cord-injured rats. Clinics (Sao Paulo).

[21] Abdelgawaad AS, Metry AB, Elnady B, El Sheriff E (2021). Anterior cervical reduction decompression fusion with plating for management of traumatic subaxial cervical spine dislocations. Global Spine J.

[22] Zhou F, Zou J, Gan M (2010). Management of fracture-dislocation of the lower cervical spine with the cervical pedicle screw system. Ann R Coll Surg Engl.

[23] Letaif OB, Damasceno ML, Cristante AF (2010). Escolha da via cirúrgica para tratamento das fraturas cervicais [Article in Portuguese]. Coluna/Columna.

[24] Miao DC, Zhang BY, Lei T, Shen Y (2017). Clinical efficacy of anterior partial corpectomy and titanium mesh fusion and internal fixation for treatment of old fracture dislocation of the lower cervical spine. Med Sci Monit.

[25] Maynard FM Jr, Bracken MB, Creasey G (1997). International standards for neurological and functional classification of spinal cord injury. Spinal Cord.

[26] Müller CW, Decker S, Thietje R, Krettek C (2013). Emergency closed reduction of a c4/5 fracture dislocation with complete paraplegia resulting in profound neurologic recovery. Case Rep Orthop.

[27] Roda JM, Castro A, Blázquez MG (1984). Hangman's fracture with complete dislocation of C-2 on C-3. Case report. J Neurosurg.

[28] Li X, Wang F, Zhang J, Hong Y, Yang Y (2020). Six-year follow-up of a survivor of cervical spine fracture and dislocation with oesophageal perforation following long scarf syndrome - a case report and literature review. BMC Musculoskelet Disord.

[29] Pinter ZW, Lawson BK, Freedman BA, Sebastian AS (2020). Atypical hangman's fracture with concomitant subaxial fracture-dislocation treated with circumferential fusion of C2-C5-a case report. Spinal Cord Ser Cases.

[30] Zan C, Zhang S, Pan S (2017). Successful surgical management of traumatic cervical spine injury in a patient with ankylosing spondylitis: a case report. Int J Clin Exp Med.

[31] Baker RP, Grubb RL Jr (1983). Complete fracture-dislocation of cervical spine without permanent neurological sequelae. J Neurosurg.

[32] Bhatia S, Sharma BS, Mathuriya SN, Pathak A, Khosla VK (1993). Complete dislocation with burst fracture of the lower cervical spine. Case report. Paraplegia.

[33] Raudenbush BL, McCalla D, Mesfin A, Rubery PT (2017). Myositis ossificans of the longus coli muscle following cervical spine fracture-dislocation. J Spinal Cord Med.

[34] Yang Y, Ma L, Li T, Liu H (2016). Redislocation after a failed surgery to treat C6/7 fracture-dislocation with pedicular fracture of the C6 vertebra: case report of a successful revision surgery, analysis of the causes, and discussion of revision surgical strategies. Medicine (Baltimore).

[35] Shiina I, Hioki S, Kamada H, Amano K, Noguchi H (2010). Treatment for lateral flexion fracture dislocation of the cervical spine: report of two cases. J Rural Med.

[36] Botolin S, VanderHeiden TF, Moore EE, Fried H, Stahel PF (2017). The role of pre-reduction MRI in the management of complex cervical spine fracture-dislocations: an ongoing controversy?. Patient Saf Surg.

